# The Influence of Sleep Quality, Vigilance, and Sleepiness on Driving-Related Cognitive Abilities: A Comparison between Young and Older Adults

**DOI:** 10.3390/brainsci10060327

**Published:** 2020-05-28

**Authors:** Chiara Bartolacci, Serena Scarpelli, Aurora D’Atri, Maurizio Gorgoni, Ludovica Annarumma, Chiara Cloos, Anna Maria Giannini, Luigi De Gennaro

**Affiliations:** 1 Department of Psychology, “Sapienza” University of Rome, 00185 Rome, Italy; chiara.bartolacci@uniroma1.it (C.B.); aurora.datri@uniroma1.it (A.D.); maurizio.gorgoni@uniroma1.it (M.G.); ludovica.annarumma@uniroma1.it (L.A.); chiara.ccloos@gmail.com (C.C.); annamaria.giannini@uniroma1.it (A.M.G.); 2 IRCCS Fondazione Santa Lucia, 00142 Rome, Italy; serena.scarpelli@uniroma1.it

**Keywords:** sleepiness, vigilance, driving abilities, aging, sleep, Vienna Test System

## Abstract

**Background**: Driving performance is strongly vulnerable to drowsiness and vigilance fluctuations. Excessive sleepiness may alter concentration, alertness, and reaction times. As people age, sleep undergoes some changes, becoming fragmented and less deep. However, the effects of these modifications on daily life have not been sufficiently investigated. Recently, the assessment of sleepiness became mandatory in Europe for people at risk who need the driving license release. Moreover, considering the expectation that people around the world are rapidly aging, it is necessary to investigate the relationships between senescence sleep changes, vigilance levels, and driving-related cognitive skills. **Method**: 80 healthy subjects (40 young adults and 40 elders) participated in the study. Sleep quality, sleepiness, and vigilance levels were assessed through the Pittsburgh Sleep Quality Index, the Karolinska Sleepiness Scale, the Epworth Sleepiness Scale, and the Psychomotor Vigilance Task (PVT). Driving-related cognitive abilities were assessed through Vienna Test System TRAFFIC, investigating selective attention, tachistoscopic perception, and risk assumption. **Results**: 2 × 2 between-subject ANOVAs showed less habitual sleep efficiency and worse performances in PVT in the older group. Unexpectedly, younger subjects show higher self-rated sleepiness. Moreover, older adults have lower performance in attention and perception tests, but they appear to be more cautious in situations involving traffic. Finally, the multiple regressions show age to be the only robust predictor of cognitive driving-related abilities. **Conclusions**: This is the first study that investigates the relationships among sleepiness/vigilance and specific driving-related cognitive skills on a sufficiently large sample. Nevertheless, the study should be considered preliminary and does not allow us to understand how specific changes in sleep architecture impact performances in the elders’ everyday life and, specifically, on driving skills.

## 1. Introduction

The World Health Organization (WHO) reported that 1.25 million people die every year due to road accidents [1] and that sleepiness is a contributing factor in 10–20% of these accidents [2,3,4]. Sleepiness represents the second most common risk factor for car accidents after alcohol consumption, resulting in many losses and high social and economic costs [5]. Excessive sleepiness at the wheel is provoked by fatigue, sleep deprivation, and changes in circadian rhythms due to shift work or the intake of sedative substances [6,7,8]. These factors may cause severe alterations in memory, concentration, alertness, and reaction times [9,10], making driving performance very vulnerable to drowsiness and vigilance fluctuations. Specifically, selective and sustained attention abilities, stimuli, risk perception, and reaction times are impaired in conditions of decreased vigilance. Vigilance is crucial for behavioral performance, as it represents an essential pre-condition for higher executive functions and appropriate response to environmental requests [11]. However, sleep intrusions (i.e., microsleep) in highly drowsy conditions increase alertness/attentiveness instability, leading to a higher number of response errors and longer reaction times [12,13,14,15,16].

According to the two-process model of sleep regulation [17,18], the modulation of sleepiness and vigilance depends on the interaction between the homeostatic factor (process S) and the circadian factor (process C). Sleep pressure expressed by process S linearly increases as a function of the previous waking hours, while it gradually decreases during the sleep period. On the other hand, process C, following a sinusoidal curve, determines a push on sleep, in relation to the time of the day. In specific moments (e.g., evening hours, early afternoon), there is a higher propensity to fall asleep, compared to other times of the day [17]. In other words, sleep restriction and fragmentation lead to homeostatic pressure, which increases during waking hours and provokes greater daytime sleepiness levels, simultaneously impacting performance levels.

As people age, sleep undergoes some macrostructural and microstructural changes [19,20,21,22]. Concerning macrostructural modifications, the senescence induces shorter sleep duration, longer sleep latency times, a higher number of intra-sleep awakenings, more time spent in the lighter stages of sleep, reduction of Rapid Eye Movement (REM) sleep and deeper stages of Non-Rapid Eye Movement (NREM) sleep, advanced phase (i.e., the anticipation of the sleep beginning and the morning awakening) and increased daytime naps (for a review, see [19]). On the other hand, microstructural changes consist of a reduction in both amplitude and density of delta waves [23,24,25,26,27], a decrease of K-complex density [28,29,30,31,32,33], a reduction in the number of slow spindles in the frontal cortex [34,35,36], and a reduction in slow-wave activity (SWA) during the aging process [23,26,37]. Furthermore, these changes occur in older individuals according to specific gender differences. Men show a larger worsening of NREM sleep and a lower Slow Wave Sleep (SWS) rebound after sleep deprivation than women [38,39]. Surprisingly, women report poorer sleep quality than men [40,41]. However, the impact of this aspect on cognitive performances is not sufficiently investigated.

Indeed, the studies about the effects of these modifications on daily life during aging are very scarce. It is still unclear whether the higher fragmentation of sleep and the depletion of SWS may increase the levels of daytime sleepiness in the healthy older adults [42,43], which would significantly impact on daily function. Some studies report more significant daytime sleepiness during aging [42,43]. Furthermore, an increase was found in the prevalence of Excessive Daytime Sleepiness (EDS) among older adults [44,45,46,47,48,49,50], which is expressed, for example, by a higher frequency of afternoon naps [51]. Moreover, drowsiness in the geriatric age is frequently underestimated [48,52,53,54,55,56] and undiagnosed [57], often considering it as if it were a normal consequence of natural aging [58]. However, studies using measures of sleepiness suggest a peculiar scenario. In fact, contrary to what one would expect, some studies showed that older subjects suffer from less sleep loss than young adults, showing good performance in sustained attention tasks [59,60,61,62]. In this respect, Lowden et al. [63] compared sleepiness in evening and night driving performances with a driving simulator in both young and older subjects. A circadian effect (time of the day) with increasing self-reported sleepiness across the sustained driving task was observed in both groups. Moreover, this study showed age differences in physiological measures, with increased power in the sigma frequency band (12–14 Hz) and higher cortisol levels in the older group. The authors speculated that the increase of cortisol levels and the higher EEG sigma power could represent protective factors against excessive sleepiness during sustained driving in the older group [63]. In their view, the excessive drowsiness while driving [64,65] and the driving errors [66] would be associated with an increase of EEG alpha (8–12 Hz) activity.

Besides studies on sleepiness, some investigations on mental workload in driving simulated performances revealed that older people are more vulnerable to the workload increase in complex scenarios than the younger subjects [67,68,69]. Specifically, older drivers showed more efforts to manage the driving performances when the complexity of the environment road scenes increased, or different procedures were simultaneously requested [68,69]. This phenomenon is probably due to the age-related decline in cognitive functions [69]. Nevertheless, older drivers revealed different compensatory skills (e.g., in the higher mental workload conditions, they would reduce speed to acquire a major control and to manage the performance) as compared to younger drivers [68].

The ability to withstand sleep deprivation/curtailment and to show normal performance, as indexed by measures of objective sleepiness, is currently a debated topic. On one side, it seems that older adults suffer less from needed sleep than young adults. On the other hand, it is still not clear whether the need for sleep decreases itself as people age [60,70,71] or whether, conversely, the older subjects are not able to generate the amount of sleep they actually need [60,70,71]. Moreover, despite the impoverishment in cognitive functions and the vulnerability in the higher mental workload situations [67,69], older adults would show efficient compensation abilities to improve their driving performance [68]. For all of these reasons, it is necessary to carry out further studies.

In Europe, an assessment for sleepiness is mandatory for high-risk individuals who apply to renew their driving license. Specifically, in Italy, a Ministerial Decree (Decreto Legge del Ministero delle Infrastrutture e dei Trasporti del 22 dicembre 2015) issues the obligation to assess sleepiness and vigilance levels in people that suffer from narcolepsy, sleep apnea, and other pathologies that could provoke EDS. Considering that by 2050 the proportion of the world’s population over 60 years will nearly double from 12% to 22% [72], it becomes crucial to clarify the possible relationships between age-related sleep changes, daytime alertness levels, and driving-related cognitive skills.

According to this background, the current study investigates the influence of sleep quality, vigilance, and sleepiness on driving-related cognitive abilities in both younger and older people to assess differences of these variables between the two age brackets (older vs. young adult groups), also considering the gender differences (male vs. female groups). Secondly, we aim to identify the sleepiness and sleep quality measures predicting the driving-related cognitive skills in the two different age brackets.

## 2. Materials and Methods

### 2.1. Subjects

Eighty healthy subjects (40 older (24 males and 16 females; mean age = 66.6 ± 5.61; range age = 58–80) and 40 young adults (21 males and 19 females; mean age = 26.2 ± 3.45; age range = 20–35)) participated in the study. We selected participants with at least two years of driving experience. Each subject was selected by a short interview to ascertain the absence of sleep disorders, disabling pathologies, drugs, or psychoactive medication assumptions (i.e., psychotropic drugs, antihistamines, alcohol, or other psychotropic substances), which can alter sleep architecture. Specifically, alcohol consumption should not exceed two alcohol units for men and one alcohol unit for women per day [73]. During the experimental session, other exclusion criteria were assessed: cognitive deterioration (Mini-Mental State Examination test scores) and psychiatric disorders (Beck Depression Inventory; State-Trait Anxiety Test 1, 2 test scores). 

Moreover, 20 of the 40 older participants were included following a previous pilot study, after which it was considered appropriate to add the driving ability assessment for tachistoscopic perception (ATAVT S1). This a posteriori addition was not able to carry out comparisons for the whole older sample and could, therefore, represent a procedural limit.

Informed consent was signed from all subjects. The study was approved by the Institutional Review Board of the Department of Psychology of the Sapienza University of Rome (#273/2019) and was conducted in accordance with the Declaration of Helsinki.

### 2.2. Measures 

Subjective sleep quality, sleepiness levels, and driving-related cognitive abilities were assessed through paper–pencil and computerized tests.

#### 2.2.1. Sleep Quality and Sleepiness Levels Measures

(a) *Pittsburgh Sleep Quality Index* (PSQI [74]) for assessing subjective sleep quality. The questionnaire investigates the sleep quality during the last month preceding the assessment. We used the Italian version of PSQI [75], with 19 items. The results are about partial scores in 7 sub-scales and a global score. The sub-scales measure subjective sleep quality (C1), sleep latency (C2), sleep duration (C3), habitual sleep efficiency (C4), sleep disturbances (C5), use of sleep medications (C6), daytime dysfunction (C7). Moreover, the questionnaire provides a measure of total sleep time (TST). A global score > 5 indicates a subjectively perceived scarce quality of sleep.
(b) *Karolinska Sleepiness Scale* (KSS [76]) is a self-report measure to assess subjective levels of state-like sleepiness. The KSS is a 9-point scale (1 = extremely alert, 3 = alert, 5 = neither alert nor sleepy, 7 = sleepy—but no difficulty remaining awake, and 9 = extremely sleepy—fighting sleep). Scores on the KSS increase with longer periods of wakefulness, and it strongly correlates with the time of the day.
(c) *Epworth Sleepiness Scale* (ESS [77]) is a self-report measure to assess subjective levels of trait-like sleepiness. The test asks to identify the probability (0 = No chance of dozing, 1 = Slight chance of dozing, 2 = Moderate chance of dozing, 3 = High chance of dozing) of falling asleep in several daily situations. Scores > 10 indicate the presence of EDS.
(d) *Psychomotor Vigilance Task* (PVT [78]) is a behavioral measure to assess sustained attention and objective levels of sleepiness. We used a 10 min version of PC-PVT software [78] for personal computers (PCs), installed on a laptop with a Windows operating system. The main dependent variables of PVT are median PVT scores, mean of 10% of the fastest reaction times (10% fastest RTs) and mean of 10% of the slowest reaction times (10% slowest RTs). The secondary variables of PVT are the lapses (RTs > 500 ms), the false starts, and the total of invalid responses.

#### 2.2.2. Driving-Related Cognitive Abilities

**Figure 1:**
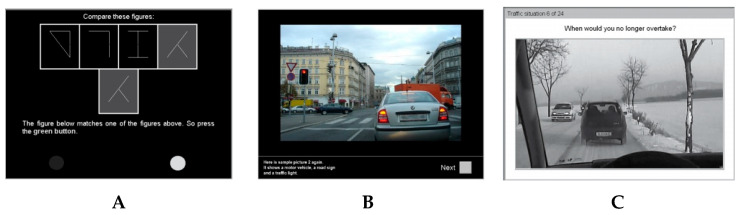
Examples of the driving-related tasks of the Vienna Test System TRAFFIC. From the left to the right: (**A**) the selective attention test (COG S/11); (**B**) the tachistoscopic perception test (ATAVT S1); (**C**) the risk-taking in the traffic test (WRB-TV).

The driving-related cognitive abilities measures are selected from the Vienna Test System TRAFFIC (Figure 1). The software has been installed on a laptop with Windows operating system. The appropriate console was used to carry out each test:

(a) *Cognitrone* (COG, Test-Set DRIVESTA). It is a selective attention assessment test that requires to compare a geometric figure with four other figures and to indicate, by pressing a button, whether among the latter there is an identical figure to the reference one (pressing the green button) or if it is not present (pressing the red button). The main dependent variable of the test is mean time of “correct rejections” (COG mean time correct rejections). This variable measures selective attention in the form of the energy required to maintain a particular level of accuracy. Since the S11 (COG/S11) version of the test was used, with flexible working time and a total of 60 items [79], the other variable of interest in the present study is the “working time” (COG total work time).
(b) *Adaptive Tachistoscopic Traffic Perception Test* (ATAVT, Test-Set DRIVESTA) evaluates the ability to obtain an overview, the skills about visual orientation, and the perceptual speed [80,81]. In other words, “obtaining an overview” is a measure of the accuracy and speed of visual observational ability and skill in gaining an overview, and of visual orientation ability. This test provides the clearest expression of perceptual capacity and speed of perception. The test’s session has a total duration of about 10 min and requires you to report, through the appropriate console, some traffic elements in a picture (pedestrians, cars, two-wheeled vehicles, road signs, and traffic lights), which is shown for a very short time frame of 1 s. The complexity level of each item is adjusted according to an adaptive gradient, keeping in mind the performance levels shown by the subject in the previous answers. In the present study, it was used the ATAVT S1 version for use in countries in which traffic drives on the right, with the steering wheel positioned on the left. The main dependent variable is “obtaining an overview”, i.e., the overall score for the task about the performance (ATAVT performance), while the secondary variable is the “working time” (ATAVT total work time) [82].
(c) *Vienna Risk-Taking Test Traffic* (WRB-TV, Test-Set PERSROAD). The test assesses the subjectively accepted risk levels by the subject. The test displays 24 videos, representing a specific traffic situation. Each video is shown the first time, in which the subject must only observe the situation and a second time, in which the subject must report (by pressing a green button) when he believes that carrying out a specific action has become too risky in the context of the shown situation. The subjectively accepted risk level is given by the lapse between pressing the button and the real danger. The dependent variable “readiness to take risks in traffic situations” (WRB-TV) is the mean time of the answers given in seconds [83].

#### 2.2.3. Assessing Measures of Psychiatric Disorders and Cognitive Deterioration

The presence of psychiatric secondaries and signs of cognitive deterioration was investigated through the Beck Depression Inventory-II, State-Trait Anxiety Inventory, and Mini-Mental State Examination.

(a) *Beck Depression Inventory-II* (BDI-II [84]). It is a self-report questionnaire consisting of 21 multiple-choice questions. The items can be divided into two sub-scales, one referring to the emotional components of depression, the other to the somatic components. Each answer provides scores from 0 to 3, which positively correlate with the severity of depressive symptoms. Total scores >13 are indicative of the presence of a depressive disorder.
(b) *State-Trait Anxiety Inventory* (STAI-Y, 1–2; [85]). It is a self-report anxiety assessment questionnaire, consisting of 40 items: 20 for the STAI-Y 1 version and 20 for the STAI-Y 2 version. The two versions evaluate state-like and trait-like anxiety. The subject is asked to indicate, choosing on a 4-point Likert scale (from nothing to very much), how much each item reflects his psycho-physical state at the time of administration. Scores ≥ 40 indicate the presence of significant anxiety levels [85].
(c) *Mini-Mental State Examination* (MMSE [86]). It is one of the most widely used tests for the rapid screening of intellectual efficiency disorders and the presence of cognitive impairment. The MMSE is made up of 30 items, referring to seven different cognitive domains: orientation in time and space, recording of words, attention, and calculation, the reenactment of words, language, and constructive praxis. The total score (between 0 and 30) is weighted for age and schooling. Scores ≤ 24 indicate the presence of cognitive impairment.

### 2.3. Procedure

The protocol administration lasted from 60 to 90 min. To consider the same circadian phase and to maintain lower levels of sleepiness, each subject carried out the assessment in a single session, between 4:00 and 7:00 p.m. Specifically, the older subjects sessions were scheduled between 4:00 and 5:30 p.m. to avoid testing them too late in the afternoon. The younger subjects were tested between 5:30 and 7:00 p.m. The administration of the tests followed this order for each participant: PSQI, ESS, PVT, KSS, WRB-TV, COG/S11, ATAVT/S1. Subsequently, all subjects filled out the self-report measures STAI-Y 1, 2, and BDI-II for psychiatric secondaries. The MMSE was administered only in older participants, for the exclusion of age-dependent cognitive impairment. 

Subjects were also asked not to alter their usual sleep habits the night before the experimental session, not to have afternoon naps and to avoid the consumption of activating substances (e.g., caffeine and chocolate) on the day of the evaluation.

### 2.4. Data Analysis

Scores were computed for each test administered in a fixed sequence (KSS; ESS; PVT; PSQI; COG/S11; ATAVT S1; WRB-TV; BDI-II; STAI-Y 1, 2; MMSE).Two-way analyses of variance (2 × 2 between-subject Age × Gender ANOVAs) were carried out for the independent group to test gender and age differences on each considered measure: subjective (KSS, ESS) and objective (PVT) sleepiness, sleep quality (PSQI), and driving variables (COG, ATAVT, WRB-TV).

Moreover, standard multiple regressions (that were all variables entered in one step) were used to assess whether driving-related abilities (i.e., COG mean time correct rejections; COG total working time; ATAVT performance; ATAVT total working time; WRB-TV) can be predicted from a set of the following independent variables (i.e., predictors): age, total sleep time (TST, obtained from the PSQI), scores in PSQI, KSS, ESS and the main variables of the PVT (median, 10% slowest RTs and 10% fastest RTs). A control for predictor gender was also led for each multiple regression model, without relevant changes as compared to these analyses.

The significance of the ANOVAs results and the multiple regressions coefficients was corrected for multiple comparisons by using a false discovery rate (FDR) [87,88].

## 3. Results

### 3.1. Age and Gender Effects

**Figure 2:**
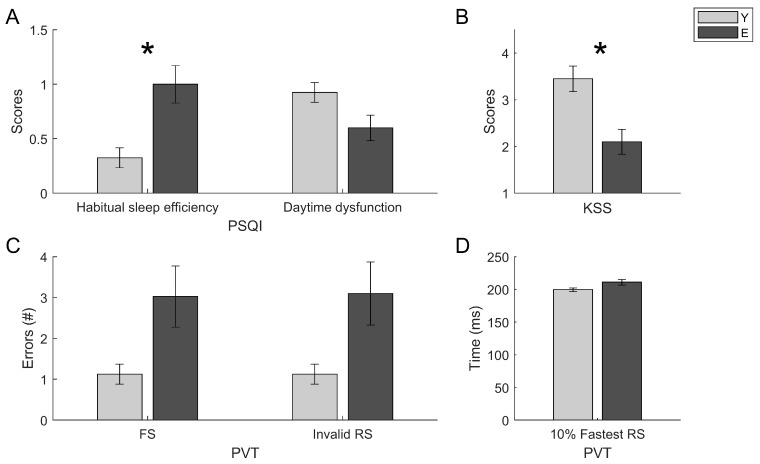
Results on measures of sleep quality and sleepiness. Means and standard errors of sleep quality (PSQI) (**A**), sleepiness (KSS) (**B**) and vigilance (PVT) measures (**C**), (**D**). For all graphs, asterisks denote significant differences.

ANOVAs on sleep quality, sleepiness, and vigilance levels show statistically significant differences in relation to the age for the following measures: habitual sleep efficiency (C4 in PSQI (F = 12.144; *p* < 0.001; ηp^2^ = 0.141)), daytime dysfunction (C7 in PSQI (F = 4.319; *p* = 0.041; ηp^2^ = 0.055)), and subjective sleepiness levels (KSS (F = 10.493; *p* = 0.001; ηp^2^ = 0.121)). No statistically significant differences were found in relation to Gender, and no Age × Gender interaction was significant (Table 1). Differences for false starts (FS (F = 5.566; *p* = 0.021; ηp^2^ = 0.070)), total invalid responses (Invalid Rs (F = 5.751; *p* = 0.019; ηp^2^ = 0.072)), and 10% fastest RTs at the PVT (F = 5.728; *p* = 0.019; ηp^2^ = 0.069) did not survive to the correction for multiple comparisons. Specifically, older subjects showed lower habitual sleep efficiency (higher scores in C4), lower subjective sleepiness levels (KSS), and more daytime dysfunction (C7) related to scarce sleep quality, as compared to the younger group (Figure 2).

**Figure 3:**
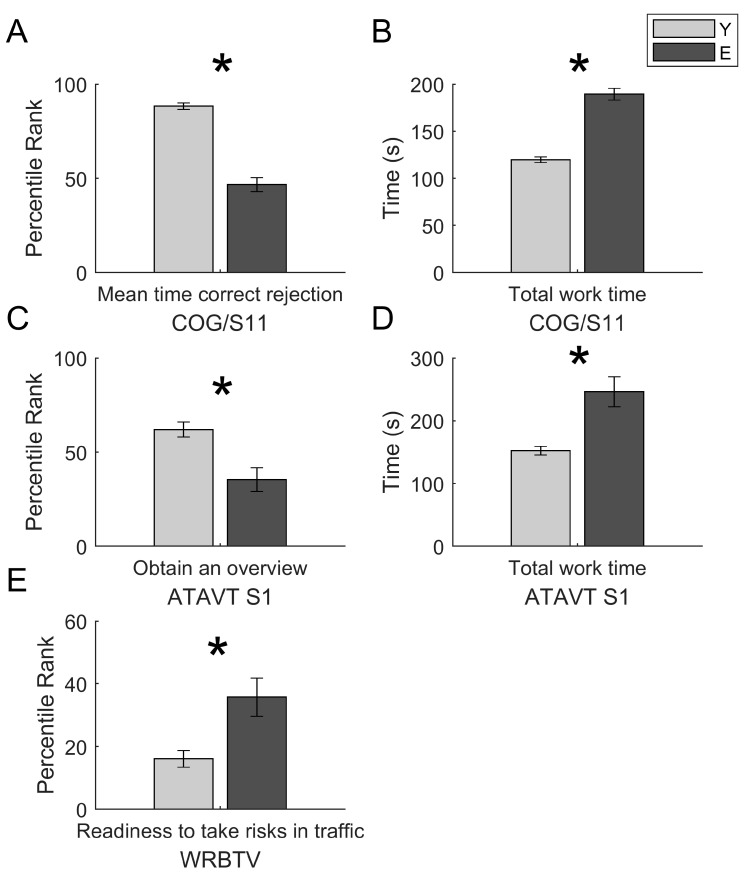
Results on measures of driving-related abilities. Means and standard errors of measures of selective attention as measured by COG S/11 (mean time correct rejections (**A**); total working time (**B**)), of tachistoscopic perception as measured by ATAVT S1 (performance (**C**); total working time (**D**)), and risk assumption (WRB-TV (**E**)). For all graphs, the asterisks denote significant differences.

ANOVAs on the measures of driving-related cognitive abilities show significant differences in relation to the age for the following measures: COG mean time of correct rejections (F = 100.947; *p* < 0.001; ηp^2^ = 0.570), COG total work time (F = 98.076; *p* < 0.001; ηp^2^ = 0.563), ATAVT performance (F = 13.207; *p* < 0.001; ηp^2^ = 0.191), ATAVT total work time (F = 22.961; *p* < 0.001; ηp^2^ = 0.291) and WRB-TV (F = 15.204; *p* < 0.001; ηp^2^ = 0.167). No statistically significant differences were found in relation to the Gender or the Age × Gender interaction (Table 2). Specifically, older subjects show longer response times in the main variable COG mean time correct rejection and worst performances in ATAVT performance. Moreover, the older group shows longer working times in both tests COG and ATAVT (Figure 3). The α-value after FDR procedure was adjusted to a critic *p* = 0.0018 [87,88]. 

### 3.2. Sleep and Drowsiness Measures as Predictors of Driving-Related Abilities

The multiple regression coefficients were always statistically significant (COG mean time correct rejections: R = 0.777, adjusted *R*^2^ = 0.559, F_8,71_ = 13.534, *p =* 0.010; COG total working time: R = 0.777, adjusted *R*^2^ = 0.559, F_8,71_ = 13.497, *p* < 0.001; ATAVT performance: R = 0.059, adjusted *R*^2^ = 0.245, F_8,51_ = 3.399, *p =* 0.003; ATAVT total working time: R = 0.640, adjusted *R*^2^ = 0.317, F_8,51_ = 4.419, *p* < 0.001; WRB-TV: R = 0.487, adjusted *R*^2^ = 0.151, F_8,71_ = 2.757, *p =* 0.010). The partial correlations indicate that age is the only predictor (among TST, PSQI, KSS, ESS, median PVT, 10% slowest RTs PVT and 10% fastest RTs PVT) entering in the multiple regression equation (Table 3). The α-value after FDR procedure was adjusted to a critic *p* = 0.01 [87,88].

In light of these results, multiple regression analyses were repeated, excluding age, to evaluate the contribution of other variables on the driving-related performances. We found that the only statistically significant multiple regression coefficient was on dependent variables COG mean time correct rejections (R = 0.438, adjusted *R*^2^ = 0.113, F_7,72_ = 2.437, *p =* 0.027) and COG total work time (R = 0.423, adjusted *R*^2^ = 0.099, F_7,72_ = 2.236, *p =* 0.041). The partial correlations in the first multiple regression indicate that PSQI, KSS, median PVT, 10% slowest RTs PVT, and 10% fastest RTs PVT are predictors. The partial correlations in the second multiple regression indicate that KSS, median PVT, 10% slowest RTs PVT, and 10% fastest RTs PVT are predictors. However, no result remains statistically significant after FDR correction (Table 4) [87,88].

An additional check for the predictor gender in the multiple regressions models is available in the supplementary material section (Tables S1 and S2).

## 4. Discussion

Our results show significant age differences in the direction of lower sleep efficiency in older as compared to the younger group, with a tendency to make more mistakes, slowing reaction times (in the 10% of fastest responses) to PVT, compatible with previous studies reporting slower reaction times in healthy older compared to the younger subjects [89]. These findings are also consistent with studies showing that sleep loss would lead to a general decrease in reaction times, which also adversely affects the range of the best performances [90]. 

Interestingly, several studies have revealed that older subjects may show less vulnerability to sleep pressure and, consequently, show a comparable or better performance than the young subjects [59,61,62]. On the other hand, young adults report an unexpected higher subjective sleepiness than the older group. Probably, the differences in participants’ lifestyles would explain this finding. Although both samples were selected with the same criteria, it should be noted that some previous studies on college students reported the presence of sleep deprivation and excessive daytime sleepiness in this specific population [91]. Higher sleepiness and disturbances during the day were consistent in younger subjects compared to the older ones [60,92]. In contrast, older adults underestimate their level of sleepiness and scarce sleep quality [57,93]. 

Furthermore, our dissociation between lower daytime sleepiness and lower sleep efficiency in the older group may be an expression of an intrinsic decline to generate and regulate sleep during healthy physiological aging (e.g., [19]). At the same time, a “floor effect” in performance tasks [61,94,95,96], may preclude evidence of larger differences in behavioral measures of diurnal vigilance. 

Concerning cognitive driving-related abilities, robust differences emerge for the age factor, without any gender difference. The older group was slower than the younger group in the selective attention test about driving skills and the tachistoscopic traffic perception test. This last result replicates a study by Kuo and Lin [97], in which the age factor negatively affected the performance and working time in the test. In this respect, we have to underline that the experimental sessions took place between the hours 4:00–7:00 p.m., a time that would favor the younger group’s performance, rather than the older ones. This aspect could represent a limitation of the study: in fact, cognitive performances are affected by the time of day [98,99]. More frequently, young people show an evening chronotype, referring to feel good and to have better performance in the late afternoon. On the other side, older people show a morning chronotype, preferring morning times for their activities [100]. Despite considering these factors important, it could partially explain the best performance of younger subjects on driving-related cognitive abilities. Although some PVT measures (i.e., a reliable and objective measure of sleepiness, sensible to circadian and homeostatic factors [12,90,101]) showed a moderate effect size, the results of the ANOVAs did not reach statistical significance. Finally, young subjects accept higher levels of risk at the wheel (WRB-TV) than older adults, consistently with the studies that report greater impulsiveness while driving by young people [102,103,104,105].

After considering the differences between the two groups, it is not surprising that the multiple regression approach confirms that age is the best predictor of poor driving performance, confirming the validity of the Vienna Test System TRAFFIC in discriminating subjects’ performances based on age [97,98,99,100,101,102,106].

The subsequent multiple regression, excluding the robust predictor of age, only points to a trend indicating vigilance, i.e., objective sleepiness (PVT), subjective state-like sleepiness (KSS), and the sleep quality (PSQI), in predicting the performance in the driving-related selective attention test, while objective and self-report sleepiness could predict the total working time. This finding suggests a potential predictive value by the PVT on a driving-related cognitive dimension, deserving further investigations.

## 5. Limitations to the Study

The current study has some limitations that should be highlighted. 

First of all, the observed dissociation between poor sleep quality and lower diurnal sleepiness in the older group needs to be confirmed with a control of sleep–wake schedule in the days preceding the experimental session and by objective measures of sleep (e.g., sleep diaries, actigraphy). This aspect did not allow us to control the participants’ compliance with the instructions received. 

Moreover, this methodological issue, along with the lack of longitudinal/repeated measures did not allow us to clearly understand if these observed age differences are determined by sleep loss (i.e., state like factors) or if they are due to specific individual differences (i.e., trait-like factors).

It is worth noting that the use of the Vienna Test System TRAFFIC is not necessarily predictive of the actual driving behavior since this instrument provides only a measure of driving-related skills. 

Finally, the fact that we considered only older people without any sleep disorder circumscribes any conclusion on the use of the PVT as a valid measure to assess sleepiness in the context of a driving license release and renewal. In other words, the assessment of subjects that are actually suffering from EDS is lacking.

## 6. Conclusions and Future Perspectives

Measurement of sleep, sleepiness, and driving-related variables make our study original, although preliminary. Our main finding concerns the age differences in driving tasks, with the older adults displaying poorer performance in attention and perception skills, while accepting minor risk than younger subjects. These results confirm the goodness and validity of the sub-scales of the Vienna Test System TRAFFIC used in our study. 

In light of the mentioned methodological limitations, we believe that future studies should be carried out (a) to assess the relation between driving-related abilities and sleepiness in older adults with sleep disturbances and/or patients with EDS; (b) to provide a comparison between sleep-deprived and normally rested subjects; (c) to provide an objective detection of sleepiness, introducing physiological measures (e.g., actigraphic or electrophysiological measures); (d) to directly explore the driving skills by specific simulated or real driving sessions.

In conclusion, we emphasize—from an applicative point of view—that the current study could represent a preliminary step toward the development of a rapid screening battery, with the purpose of a driving license release and renewal. Considering our “aging society”, we recommend the introduction of validated assessment tools for detecting excessive diurnal sleepiness.

## Figures and Tables

**Table 1:** Results of 2 × 2 between-subjects Age × Gender ANOVAs on sleep quality (PSQI), sleepiness (KSS), and vigilance (PVT) measures. The table shows Fisher coefficients (F), the associated probability (*p* < 0.0018), and the effect size (partial eta-squared, ηp^2^).

Variables	Groups	F	*p*	ηp^2^
C4 PSQI	Age	12.144	***p* < 0.001**	0.141
	Gender	0.051	0.822	0.001
	Age × Gender	0.652	0.412	0.009
C7 PSQI	Age	4.319	0.041	0.055
	Gender	1.639	0.206	0.022
	Age × Gender	0.101	0.752	0.001
KSS	Age	10.493	**0.001**	0.121
	Gender	0.659	0.419	0.009
	Age × Gender	3.025	0.086	0.038
False Starts PVT	Age	5.566	0.021	0.070
	Gender	0.125	0.724	0.002
	Age × Gender	0.182	0.67	0.002
Invalid Responses PVT	Age	5.751	0.019	0.072
	Gender	0.053	0.817	0.001
	Age × Gender	0.275	0.601	0.004
10% Fastest RTs PVT	Age	5.728	0.019	0.069
	Gender	0.71	0.402	0.009
	Age × Gender	0.623	0.432	0.008

Bold—mark the statistically significant result.

**Table 2:** The 2 × 2 results of 2 × 2 between-subjects Age × Gender ANOVAs on the measures of selective attention (COG mean time correct rejections and total working time), of tachistoscopic perception (ATAVT tachistoscopic perception and total working time) and risk assumption (WRB-TV). The table shows Fisher coefficients (F) the associated probability (*p* < 0.0018), and the effect size (partial eta-squared, ηp^2^).

Variables	Groups	F	*p*	ηp^2^
COG mean time correct rejection	Age	100.947	***p*** **< 0.001**	0.570
	Gender	0.003	0.956	0.00004
	Age × Gender	0.628	0.431	0.008
COG total working time	Age	98.076	***p*** **< 0.001**	0.563
	Gender	0.596	0.442	0.008
	Age × Gender	0.164	0.686	0.002
ATAVT performance	Age	13.207	***p*** **< 0.001**	0.191
	Gender	2.010	0.162	0.035
	Age × Gender	2.143	0.149	0.037
ATAVT total working time	Age	22.961	***p*** **< 0.001**	0.291
	Gender	0.001	0.991	0.000002
	Age × Gender	0.325	0.571	0.006
WRBTV	Age	15.204	***p*** **< 0.001**	0.167
	Gender	0.001	0.982	0.000007
	Age × Gender	0.106	0.746	0.001

Bold—mark the statistically significant result.

**Table 3:** Results of multiple regressions (*p* < 0.01), considering driving-related cognitive abilities (COG, ATAVT, WRB-TV) as criterion variables and age, TST, PSQI, KSS, ESS, Median PVT, 10% slowest RTs PVT, 10% fastest RTs PVT as predictors.

Dependent Variables	Predictors	Beta	Coefficients of Partial Correlation	*t*	*p*-Level
**COG mean time correct rejections** R = 0.777; adjusted *R*^2^ = 0.559;$\;F_{8,71^{}}=$ 13.534; ***p* < 0.001**	Age	−0.757	−0.714	−8.599	***p* < 0.001**
	TST	0.113	0.171	1.465	0.147
	PSQI	−0.098	−0.136	−1.156	0.251
	KSS	−0.029	−0.040	−0.337	0.737
	ESS	−0.047	−0.069	−0.586	0.560
	Median PVT	0.038	0.019	0.158	0.875
	10% slowest RTs PVT	−0.078	−0.065	−0.546	0.586
	10% fastest RTs PVT	0.010	0.007	0.057	0.954
**COG total working time** R = 0.777; adjusted *R*^2^ = 0.559;$\;F_{8,71^{}}=$ 13.497; ***p* < 0.001**	Age	0.768	0.719	8.719	***p* < 0.001**
	TST	−0.108	−0.163	−1.391	0.169
	PSQI	0.060	0.084	0.711	0.479
	KSS	0.036	0.051	0.429	0.669
	ESS	0.024	0.035	0.299	0.766
	Median PVT	0.003	0.001	0.011	0.991
	10% slowest RTs PVT	0.089	0.074	0.622	0.536
	10% fastest RTs PVT	−0.043	−0.028	−0.233	0.816
**ATAVT performance** R = 0.059; adjusted *R*^2^ = 0.245;$\;F_{8,51^{}}=$ 3.399; ***p* = 0.003**	Age	−0.543	−0.513	−4.272	***p* < 0.001**
	TST	0.173	0.197	1.432	0.158
	PSQI	−0.010	−0.011	−0.078	0.938
	KSS	−0.106	−0.114	−0.817	0.418
	ESS	−0.015	−0.017	−0.123	0.902
	Median PVT	−0.359	−0.171	−1.238	0.221
	10% slowest RTs PVT	0.024	0.018	0.127	0.900
	10% fastest RTs PVT	0.044	0.029	0.205	0.838
**ATAVT total working time** R = 0.640; adjusted *R*^2^ = 0.317;$\;F_{8,51^{}}=$ 4.419; ***p* < 0.001**	Age	0.602	0.571	4.971	***p* < 0.001**
	TST	0.054	0.066	0.471	0.640
	PSQI	−0.138	−0.165	−1.191	0.239
	KSS	−0.070	−0.079	−0.568	0.572
	ESS	−0.083	−0.100	−0.715	0.478
	Median PVT	0.193	0.098	0.700	0.487
	10% slowest RTs PVT	0.033	0.026	0.184	0.854
	10% fastest RTs PVT	−0.349	−0.233	−1.713	0.093
**WRB-TV** R = 0.487; adjusted *R*^2^ = 0.151;$\;F_{8,71^{}}=$ 2.757; ***p* = 0.010**	Age	0.384	0.350	3.145	**0.002**
	TST	−0.024	−0.027	−0.227	0.821
	PSQI	0.061	0.061	0.516	0.607
	KSS	−0.032	−0.033	−0.274	0.785
	ESS	−0.160	−0.168	−1.434	0.156
	Median PVT	0.118	0.041	0.350	0.727
	10% slowest RTs PVT	−0.320	−0.189	−1.621	0.109
	10% fastest RTs PVT	0.067	0.031	0.265	0.792

Bold—mark the statistically significant result.

**Table 4:** Results of the multiple regression considering the performance and the working time in the selective attention test (COG mean time correct rejection and COG total work time) as criterion variable and TST, PSQI, KSS, ESS, Median PVT, 10% slowest RTs PVT, 10% fastest RTs PVT as predictors. After the correction for multiple comparisons by using FDR, the results do not remain statistically significant.

Dependent Variables	Predictors	Beta	Coefficients of Partial Correlation	*t*	*p*-Level
**COG mean time correct rejections** R = 0.438; adjusted *R*^2^ = 0.113;$\;F_{7,72^{}}=$ 2.437; *p* = 0.027	TST	0.004	0.004	0.033	0.973
	PSQI	−0.254	−0.248	−2.170	0.033
	KSS	0.231	0.236	2.057	0.043
	ESS	−0.006	−0.006	−0.049	0.961
	Median PVT	0.707	0.246	2.158	0.034
	10% slowest RTs PVT	−0.390	−0.229	−2.000	0.049
	10% fastest RTs PVT	−0.501	−0.234	−2.042	0.045
**COG total working time** R = 0.423; adjusted *R*^2^ = 0.099;$\;F_{7,72^{}}=$ 2.236; *p* = 0.041	TST	0.004	0.004	0.034	0.973
	PSQI	0.219	0.214	1.855	0.068
	KSS	−0.227	−0.230	−2.005	0.049
	ESS	−0.018	−0.019	−0.158	0.875
	Median PVT	−0.675	−0.234	−2.046	0.044
	10% slowest RTs PVT	0.406	0.236	2.063	0.043
	10% fastest RTs PVT	0.477	0.221	1.926	0.058

## Supplementary Materials

The following are available online at https://www.mdpi.com/2076-3425/10/6/327/s1. Table S1: Results of multiple regression model with the control for gender; Table S2: Results of multiple regression model with the control for gender, excluding the age predictor.
