# Supplementary material for: The Influence of Sleep Quality, Vigilance, and Sleepiness on Driving-Related Cognitive Abilities: A Comparison between Young and Older Adults
Source: Brain Sci. 2020 May 28;10(6):327. doi: 10.3390/brainsci10060327 (PMC7349304; doi:10.3390/brainsci10060327)
Supplement: Supplementary file 1 [file brainsci-10-00327-s001.pdf]

Supplementary Materials: The Influence of Sleep Quality, Vigilance, and Sleepiness on Driving-Related Cognitive Abilities: A Comparison between Young and Older Adults

Table S1. Results of multiple regression model with the control for gender. Results of multiple regressions (the α-value after FDR procedure was adjusted to a critic *p=0.019*) considering driving-related cognitive abilities (COG, ATAVT, WRB-TV) as criterion variables and gender, age, TST, PSQI, KSS, ESS, Median PVT, 10% slowest RTs PVT, 10% fastest RTs PVT as predictors. After the correction for multiple comparisons by using FDR, each result remains statistically significant.

**Dependent Variables**	**Predictors**	**Beta**	**Coefficients of Partial Correlation**	***t***	***p*-level**
	Gender	-0.024	-0.036	-0.304	0.762
	Age	-0.758	-0.715	-8.549	***p*<0.001**
**COG mean time**	PSQI	-0.099	-0.138	-1.165	0.248
**correct rejections**	TST	0.114	0.173	1.466	0.147
R= 0.777; adjusted	KSS	-0.026	-0.037	-0.310	0.758
R^2=0.554; $F_{9.70}$ =11.887;	ESS	-0.046	-0.068	-0.569	0.571
***P*<0.001**	Median PVT	0.053	0.026	0.214	0.831
	10% slowest RTs PVT	-0.084	-0.069	-0.580	0.564
	10% fastest RTs PVT	0.003	0.002	0.018	0.986
	Gender	-0.036	-0.056	-0.466	0.643
	Age	0.767	0.719	8.653	***p*<0.001**
**COG total working**	PSQI	0.058	0.081	0.679	0.499
**time**	TST	-0.106	-0.161	-1.366	0.176
R=0.778; adjusted	KSS	0.039	0.055	0.462	0.645
R^2=0.554; $F_{9.70}$ =11.889;	ESS	0.026	0.038	0.316	0.753
***P*<0.001**	Median PVT	0.026	0.012	0.103	0.918
	10% slowest RTs PVT	0.079	0.065	0.548	0.585
	10% fastest RTs PVT	-0.054	-0.035	-0.289	0.773
	Gender	-0.240	-0.286	-2.111	**0.040**
**ATAVT performance**	Age	-0.552	-0.535	-4.483	***p*<0.001**
R=0.633; adjusted	PSQI	-0.014	-0.016	-0.116	0.908
R^2=0.293; $F_{9.50}$ =3.722;	TST	0.191	0.224	1.625	0.111
***P*=0.001**	KSS	-0.089	-0.100	-0.710	0.481
	ESS	0.014	0.017	0.120	0.905
	Median PVT	-0.247	-0.121	-0.865	0.391
	10% slowest RTs PVT	-0.002	-0.002	-0.011	0.991
	10% fastest RTs PVT	-0.024	-0.017	-0.117	0.908
	Gender	-0.029	-0.036	-0.255	0.800
**ATAVT total working**	Age	0.601	0.571	4.914	***p*<0.001**
**time**	PSQI	-0.139	-0.165	-1.184	0.242
	TST	0.056	0.068	0.483	0.631

R=0.640; adjusted	KSS	-0.068	-0.077	-0.546	0.588
R^2=0.304; $F_{9.50}$ =3.863;	ESS	-0.079	-0.095	-0.673	0.504
***P*=0.001**	Median PVT	0.207	0.103	0.729	0.469
	10% slowest RTs PVT	0.030	0.023	0.165	0.869
	10% fastest RTs PVT	-0.357	-0.236	-1.716	0.092
	Gender	0.018	0.020	0.168	0.867
**WRB-TV**	Age	0.385	0.350	3.127	**0.003**
R=0.487; adjusted	PSQI	0.062	0.062	0.522	0.603
R^2=0.139; $F_{9.70}$ =2.420;	TST	-0.025	-0.028	-0.231	0.818
***P*=0.019**	KSS	-0.034	-0.034	-0.285	0.776
	ESS	-0.160	-0.169	-1.430	0.157
	Median PVT	0.107	0.037	0.307	0.760
	10% slowest RTs PVT	-0.315	-0.185	-1.571	0.121
	10% fastest RTs PVT	0.073	0.034	0.283	0.778

Table S2. Results of multiple regression model with the control for gender, excluding the age predictor. Results of the multiple regression considering the performance in the selective attention test (COG mean time correct rejection) as criterion variable and gender, TST, PSQI, KSS, ESS, Median PVT, 10% slowest RTs PVT, 10% fastest RTs PVT as predictors. After the correction for multiple comparisons by using FDR, the result does not remain statistically significant.

**Dependent Variables**	**Predictors**	**Beta**	**Coefficients of Partial Correlation**	***t***	***p*-level**
	Gender	-0.004	-0.005	-0.040	0.968
**COG mean time**	PSQI	-0.254	-0.248	-2.153	0.035
**correct rejections**	TST	0.004	0.004	0.035	0.973
R= 0.438; adjusted	KSS	0.231	0.235	2.037	0.045
R^2=0.100; $F_{8.71}$ =2.103;	ESS	-0.005	-0.006	-0.046	0.963
***P*=0.047**	Median PVT	0.710	0.242	2.101	0.039
	10% slowest RTs PVT	-0.391	-0.228	-1.970	0.053
	10% fastest RTs PVT	-0.503	-0.232	-2.012	0.048
